# Development of a capture sequencing assay for enhanced detection and genotyping of tick-borne pathogens

**DOI:** 10.1038/s41598-021-91956-z

**Published:** 2021-06-11

**Authors:** Komal Jain, Teresa Tagliafierro, Adriana Marques, Santiago Sanchez-Vicente, Alper Gokden, Brian Fallon, Nischay Mishra, Thomas Briese, Vishal Kapoor, Stephen Sameroff, Cheng Guo, Luis A. Marcos, Linden Hu, W. Ian Lipkin, Rafal Tokarz

**Affiliations:** 1 Center for Infection and Immunity, Mailman School of Public Health, Columbia University, New York, NY USA; 2 Laboratory of Clinical Immunology and Microbiology, National Institute of Allergy and Infectious Diseases, National Institutes of Health, Bethesda, MD USA; 3 Department of Psychiatry, Columbia University, New York, NY USA; 4 Department of Epidemiology, Mailman School of Public Health, Columbia University, New York, NY USA; 5 Department of Medicine (Division of Infectious Diseases), Department of Microbiology and Immunology, State University of New York at Stony Brook, NY Stony Brook, USA; 6 Department of Molecular Biology and Microbiology, Tufts University, Boston, MA USA

**Keywords:** Infectious-disease diagnostics, Next-generation sequencing

## Abstract

Inadequate sensitivity has been the primary limitation for implementing high-throughput sequencing for studies of tick-borne agents. Here we describe the development of TBDCapSeq, a sequencing assay that uses hybridization capture probes that cover the complete genomes of the eleven most common tick-borne agents found in the United States. The probes are used for solution-based capture and enrichment of pathogen nucleic acid followed by high-throughput sequencing. We evaluated the performance of TBDCapSeq to surveil samples that included human whole blood, mouse tissues, and field-collected ticks. For *Borrelia burgdorferi* and *Babesia microti*, the sensitivity of TBDCapSeq was comparable and occasionally exceeded the performance of agent-specific quantitative PCR and resulted in 25 to > 10,000-fold increase in pathogen reads when compared to standard unbiased sequencing. TBDCapSeq also enabled genome analyses directly within vertebrate and tick hosts. The implementation of TBDCapSeq could have major impact in studies of tick-borne pathogens by improving detection and facilitating genomic research that was previously unachievable with standard sequencing approaches.

## Introduction

Early detection is critical for prompt and effective treatment of acute infectious diseases. Molecular assays are the optimal method for early and rapid detection of pathogenic agents. For tick-borne diseases (TBD), the lack of accurate early diagnosis can result in delayed treatment, augment morbidity, and increase the likelihood of developing persistent symptoms^1,2^. For some agents of TBD, such as *Anaplasma phagocytophilum* and *Babesia microti*, molecular diagnostic assays are highly useful^3^. For diagnosis of Lyme disease, however, the low sensitivity of molecular assays in the majority of clinical presentations has prevented their extensive implementation as an effective diagnostic tool^4–7^. The exception is synovial fluid in patients with Lyme arthritis, where PCR has 70 to 85% sensitivity^8,9^. The primary challenges in molecular diagnoses of *Borrelia burgdorferi* sensu stricto (s.s.) infection include low pathogen burden coupled with transient spirochetaemia^10,11^. As a result, serology remains the primary means of diagnosis for Lyme disease and the majority of TBD^11–13^. There is nonetheless a need for molecular assays for differential diagnosis of TBD that can complement serology.

PCR and unbiased high-throughput sequencing (UHTS) are the primary molecular assays employed for detection of infectious agents^14,15^. The advantages of UHTS over PCR include the lack of dependence for analogous primer and template sequences, the capacity to detect all agents, and the ability to accurately identify novel species or strains^15–20^. However, PCR generally retains an advantage in sensitivity, cost and simplicity. The sensitivity of UHTS can be enhanced with deeper sequencing, but this approach is currently financially unfeasible. The recent implementation of capture-based sequencing assays has had a major impact on the utility of high-throughput sequencing (HTS) for pathogen detection^21–23^. This approach uses agent-specific probes to selectively capture the template of interest prior to sequencing and results in a remarkable increase in sequencing reads for the captured template when compared to UHTS. Our Center has developed probe-based capture assays for viruses (VirCapSeq) and bacteria (BacCapSeq) that result in assay sensitivity equal or greater to real-time PCR^24,25^. Dual barcoding enables simultaneous testing of 50 samples in a single run without compromising sensitivity and providing cost efficiency. Thus, employment of capture assays would provide substantial improvement in detection of tick-borne agents over UHTS and PCR. In addition, the far greater capacity of capture-sequencing assays to generate complete genome sequences relative to UHTS can lead to increased understanding of how strain diversity impacts disease. For *B. burgdorferi* s.s*.*, work in animal models has implicated several strains with increased likelihood of systemic dissemination^26^. In particular, plasmid content and specific OspC types have been associated with enhanced pathogenesis^27–30^. Different strains within *B. burgdorferi* s.s*.* can be distinguished that impact pathogenicity and virulence in humans^26,31,32^. *B. burgdorferi* s.s. RST1 strains, which appear to account for approximately 40% of Lyme borreliosis cases in the northeastern US, are more likely to disseminate hematogenously and are associated with a higher risk of antibiotic-refractory Lyme arthritis^33,34^. Similarly, certain variations in OspC have been shown to be associated with disseminated infection in humans^31,35^. Unfortunately, assay limitations have prevented comprehensive genomic analyses of *B. burgdorferi* s.s. in human specimens and, at present, there is a great paucity of *B. burgdorferi* s.s. genomic data obtained directly from patient samples. This limits our understanding of how strain diversity could influence the development of Lyme disease-associated syndromes such as neuroborreliosis, Lyme arthritis and post-treatment Lyme disease syndrome. In this work, we address the need for diagnostic improvement combined with genetic typing with the development of Tick-Borne Disease Capture Sequencing assay (TBDCapSeq). We demonstrate that TBDCapSeq is capable of enhanced targeted detection of all major agents of TBD found in the United States that also provides invaluable genomic data that can be used to augment our understanding of strain variation and its importance to tick-borne disease.

## Results

To assess the performance of TBDCapSeq, we selected pathogen-infected ticks (larvae, nymphs and adults), mouse tissues, human whole blood, as well as controls (Table 1). These samples were available from other published or ongoing studies, and provided an opportunity to evaluate TBDCapSeq on different sample matrices^36–38^. For this study, we primarily focused on samples that were infected with *B. burgdorferi* s.s. or *B. microti*, as these agents are among the most frequent causes of tick-borne illness in the US.

**Table 1:** List of samples analyzed by TBDCapSeq.

Experiment	Sample type (N)	Number of samples
1	**Mouse tissues**; heart (3) ear (3) bladder (3); **larval ticks** (2)	11^a^
2	**Mouse tissues**; heart (3) ear (3) bladder (3) ankle (3); **larval ticks** (2)	14^a^
3	**Ticks**; *Ixodes scapularis* (16)*, Amblyomma americanum* (2 pools), *Dermacentor variabilis* (3)	20
4	**Whole blood**; *Babesia microti* contrived samples (16); healthy controls (2)	18
5	**Whole blood;** Babesiosis (19); healthy control (1); salmon sperm (1)	21
6	**Whole blood;** acute Lyme disease (15); acute babesiosis (3); healthy control (1)	19
7	**Whole blood**; *Borrelia burgdorferi* contrived samples (10); healthy controls (2)	12

^a^Each sample was analyzed by unbiased high-throughput sequencing and TBDCapSeq.

### Mouse tissues

To evaluate the utility of TBDCapSeq with respect to UHTS for testing tissue samples, we examined heart, ear, and bladder tissues from three C3H mice infected with 10^5^ spirochetes of the N40 D10E9 strain of *B. burgdorferi* s.s. (Table 2). All mice were culture positive. We also tested two replete larval ticks that fed on these mice. Prior to sequencing, we used an *ospA* qPCR assay to estimate the *B. burgdorferi* s.s*.* burden for all samples. In murine tissues, the Ct ranged from 29.48 to 35.49 (corresponding to approximately 2500 to 25 copies of *ospA*). The quantity of *B. burgdorferi* s.s. in the ticks was > 100 fold higher, with Cts of 21.17 and 22.25 (both > 5 × 10^5^). All 11 samples were sequenced together in two pools on a single Illumina flow cell. One pool was subjected to capture with the TBDCapSeq probes. The second pool was sequenced using a standard unbiased approach. We observed a substantial increase in the number of *B. burgdorferi* s.s*.* reads using TBDCapSeq when compared to UHTS. After normalization, the increase in *B. burgdorferi* s.s*.* reads in murine tissues with TBDCapSeq ranged from 1133-fold to 9332-fold. We also observed a sizable increase in *B. burgdorferi* s.s*.* reads from the larval ticks with TBDCapSeq (125-fold and 140-fold).

**Table 2:** Read enrichment using TBDCapSeq compared to unbiased high-throughput sequencing (UHTS) in experiment 1.

Sample type	*ospA* Ct	Normalized reads (*Borrelia* reads per million total reads)		Fold enrichment of reads with TBDCapSeq over UHTS
		TBDCap seq	UHTS	
Replete larval tick	21.17	544,328	5707	140
Replete larval tick	22.25	323,316	2578	125
Mouse 1, Heart	33.62	1272	0.067	1890
Mouse 1, Ear	31.40	20,819	7.27	2863
Mouse 1, Bladder	32.62	2930	0.35	8338
Mouse 2, Heart	34.23	4790	1.83	2615
Mouse 2, Ear	29.64	34,353	13	2648
Mouse 2, Bladder	35.49	392	0.34	1133
Mouse 3, Heart	33.47	2009	0.062	3213
Mouse 3, Ear	29.48	3546	1.01	3494
Mouse 3, Bladder	ND^a^	829	0.09	9332

^a^ND = not done.

**Figure 1:**
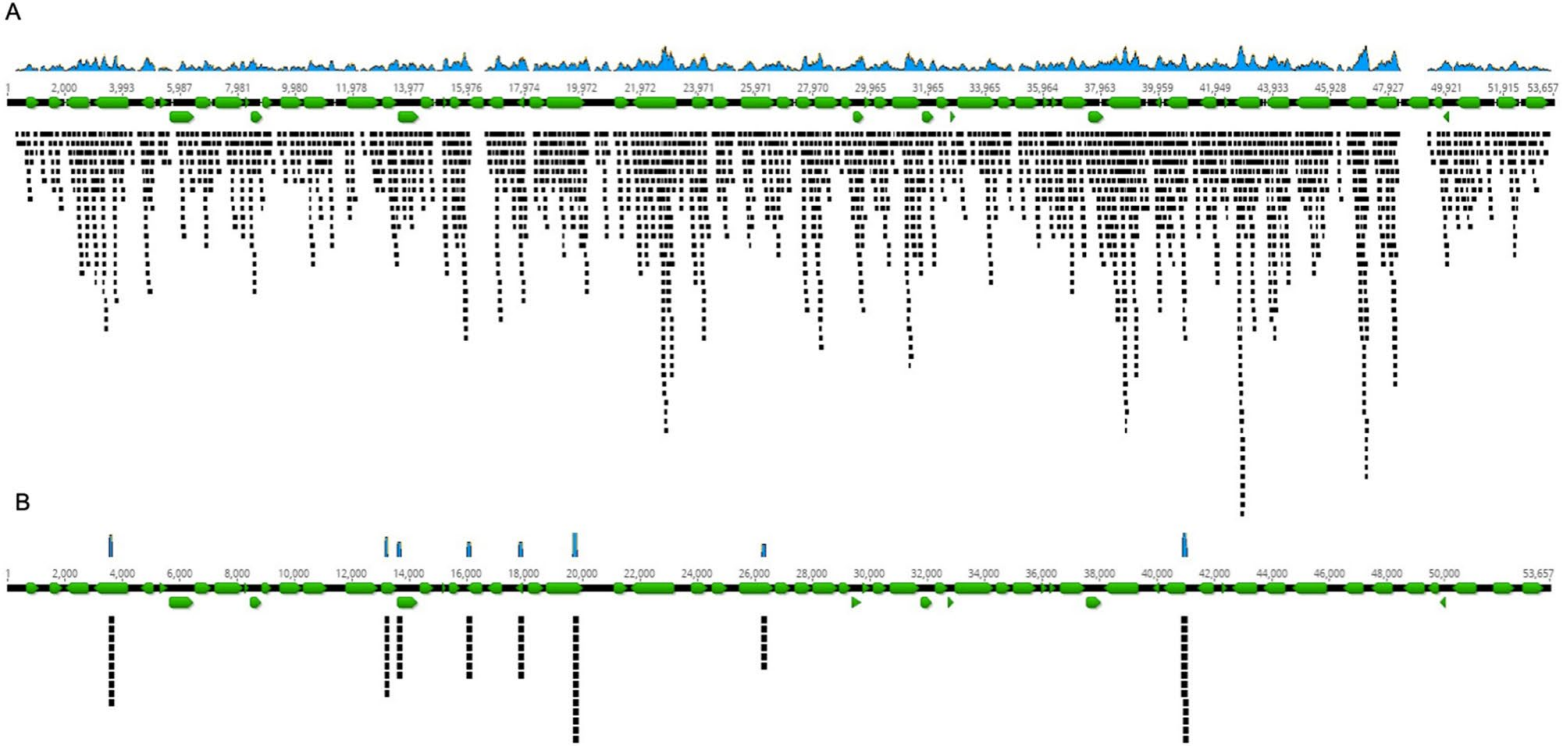
Mapping of *B. burgdorferi* s.s. reads obtained by TBDCapSeq to the Lp54 plasmid. All quality filtered reads were mapped directly to the B31 Lp54 sequence (accession number NC_001857, www.ncbi.nlm.nih.gov). (**A**) reads obtained from the bladder tissue of mouse 3. (**B**) reads from the bladder tissue of mouse 1. The sample was negative by qPCR and UHTS. The black horizontal line represents the contiguous linear length of the plasmid with the numbers representing the nucleotide positions within the plasmid. Green arrows represent open reading frames. Assemblies were performed in Geneious v 10.0.9 (www.geneious.com).

Next, we examined tissues from three C57BL/6 mice (one mouse infected with *B. burgdorferi* s.s*.* N40 D10E9, one mouse infected with *B. burgdorferi* s.s*.* B31, and one mouse infected with both N40 D10E9 and B31) and two replete larval ticks (one tick infected with *B. burgdorferi* s.s*.* B31, and a negative control). Tick and mouse samples were processed blinded as to their infection status in this experiment, since our primary aim was to demonstrate the improvements in detection and strain identification achieved by TBDCapSeq. Samples were sequenced on two separate flow cells, one using UHTS and another using TBDCapSeq. We also determined B. burgdorferi s.s. burden by qPCR. Again, TBDCapSeq generated a remarkable increase in reads with TBDCapSeq relative to UHTS (Table 3). In murine samples with detectable *B. burgdorferi* s.s*.* reads using both sequencing approaches, we obtained a 6550-fold to 52,000-fold enrichment using TBDCapSeq over UHTS. In three murine samples with a low bacterial load (Cts 35 to 37, corresponding to approximately 25 to 6 *ospA* copies), two samples did not generate any *Borrelia* reads with UHTS, and the third produced only a single *Borrelia* read. With TBDCapSeq, we generated between 9817 and 26,671 *Borrelia* reads for these samples. One sample (bladder tissue-mouse 1) was negative by both qPCR and UHTS. With TBDCapSeq, we obtained 781 non-ribosomal reads (out of 1329), originating from the chromosome and multiple linear and circular plasmids. For example, we obtained 74 reads from 8 regions within the lp54 plasmid, accounting for 1005 nt, or 2.1% of the total plasmid sequence (Fig. 1). We also obtained a notable improvement in genome recovery (Table 4). In samples with a qPCR Ct of approximately 35, we were able to recover nearly 20% of the *B. burgdorferi* s.s. genome. In two samples with a higher *B. burgdorferi* s.s. burden (qPCR Cts between 30 and 31, corresponding to approximately 2000 to 1000 *ospA* copies), *B. burgdorferi* s.s*.* reads accounted for > 9.5% of the total reads generated by TBDCapSeq, and we were able to assemble > 95% of each genomic segment. For the two larval ticks, we assembled the complete *B. burgdorferi* s.s. genome for tick 1. The other tick did not yield any *Borrelia* reads, in agreement with the corresponding *ospA*-negative qPCR data.

**Table 3:** Read enrichment using TBDCapSeq compared to unbiased sequencing in experiment 2.

*ospA* Ct	Sample type	Sequencing type	Total reads	# of reads mapped to B31	% of reads mapped to B31	Normalized reads (*Borrelia* reads per million total reads)	Fold enrichment
35.15	Heart Tissue- Mouse 1	Unbiased	10,473,737	0	0	0	1603.10
		TBDCAPSEQ	13,922,800	22,319	0.18	1603.10	
34.06	Ear Tissue- Mouse 1	Unbiased	11,451,201	22	0	1.921	6550.90
		TBDCAPSEQ	11,160,954	140,453	1.4	12,584.30	
33.99	Ankle Tissue- Mouse 1	Unbiased	9,663,953	3	0	0.31	52,212.80
		TBDCAPSEQ	2,454,532	39,729	1.938	16,185.98	
ND	Bladder Tissue- Mouse 1	Unbiased	8,675,559	0	0	0	101.3
		TBDCAPSEQ	13,120,042	1329	0.01	101.3	
31	Heart Tissue- Mouse 2	Unbiased	8,956,637	32	0	0	46,207.90
		TBDCAPSEQ	12,485,504	576,929	5.06	46,207.90	
33.38	Ear Tissue- Mouse 2	Unbiased	10,377,529	35	0	3.37	8804.90
		TBDCAPSEQ	11,411,883	338,619	3.27	29,672.50	
30.49	Ankle Tissue- Mouse 2	Unbiased	7,332,169	46	0.001	6.27	13,893
		TBDCAPSEQ	10,915,727	950,856	9.5	87,108.80	
36.67	Bladder Tissue- Mouse 2	Unbiased	10,477,826	1	0	0.1	6691
		TBDCAPSEQ	14,041,858	9817	0.08	699.1	
30.29	Heart Tissue- Mouse 3	Unbiased	8,959,037	131	0.002	14.62	1137.03
		TBDCAPSEQ	13,331,701	2,141,384	17.46	160,623.50	
32.2	Ear Tissue- Mouse 3	Unbiased	8,148,936	37	0.001	4.54	10,938.30
		TBDCAPSEQ	13,089,033	650,000	5.52	49,659.90	
32.83	Ankle Tissue- Mouse 3	Unbiased	7,253,259	18	0	2.48	10,517.70
		TBDCAPSEQ	11,386,644	297,007	2.88	26,083.80	
36.32	Bladder Tissue- Mouse 3	Unbiased	8,663,429	0	0	0	2466.70
		TBDCAPSEQ	10,812,361	26,671	0.27	2466.70	
24.03	Tick (fed larva 1)	Unbiased	7,014,321	7657	0.122	1091.62	68.89
		TBDCAPSEQ	64,314,285	4,836,678	8.129	75,203.79	
ND^c^	Tick (fed larva 2)	Unbiased	8,210,936	0	0	0	0
		TBDCAPSEQ	63,077,105	1335^a^	0	0	
ND	Salmon sperm DNA	Unbiased	8,214,254	0	0	0	0
		TBDCAPSEQ	694,238	31^b^	0	0	

^a^All mapped to 16S and 23S rRNA of *Rickettsia buchneri.*

^b^All represented non-*Borrelia* 16S rRNA reads.

^c^ND = not done.

**Table 4:** Genome recovery of *B. burgdorferi* in murine tissues with TBDCapSeq.

Accession number	Chromosome/plasmid	% coverage Mouse 1, Heart (qPCR Ct 35.15)	% coverage Mouse 2, Heart (qPCR Ct 31)	% coverage Mouse 3, Heart (qPCR Ct 30.29)
NC_000948	cp32-1	51.73	98.92	99.63
NC_000949	cp32-3	47.17	99.24	99.27
NC_000950	cp-32-4	53.49	99.47	100.00
NC_000951	cp32-6	56.23	99.34	99.35
NC_000952	cp32-7	48.81	97.98	99.96
NC_000953	cp32-8	51.28	98.99	99.56
NC_000954	cp32-9	56.18	99.14	99.60
NC_000955	lp21	1.22	99.87	100.00
NC_000956	lp56	34.23	98.69	99.49
NC_000957	lp5	2.70	100.00	100.00
NC_001318	CHR	20.42	97.93	98.55
NC_001849	lp17	35.41	99.67	99.72
NC_001850	lp25	21.83	94.77	98.54
NC_001851	lp28-1	0.89	100.00	99.63
NC_001852	lp28-2	18.49	98.34	98.90
NC_001853	lp28-3	15.71	95.97	99.18
NC_001854	lp28-4	16.78	97.97	98.22
NC_001855	Lp36	5.21	99.36	99.24
NC_001856	lp38	2.18	97.56	99.03
NC_001857	lp54	38.08	99.54	99.58
NC_001903	cp26	19.39	98.80	98.18
NC_001904	cp9	5.51	17.47	22.28

**Figure 2:**
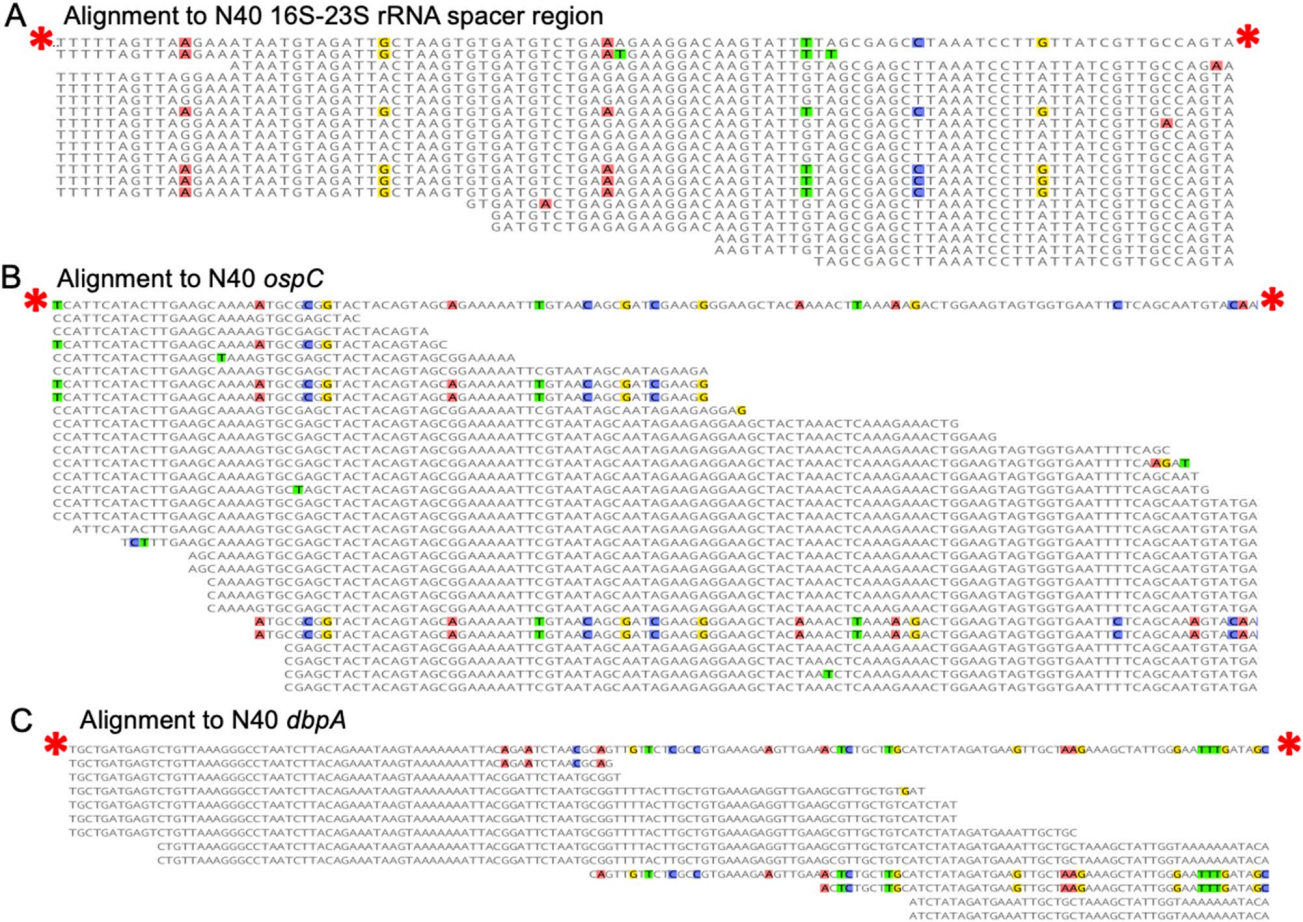
Identification of an infection with multiple strains of *B. burgdorferi* s.s. by TBDCapSeq. Shown are alignments of TBDCapSeq reads to fragments of (**A**) 16S-23S rRNA spacer region, (**B**) *dbpA*, and (**C**) *ospC* from the N40 strain (accession numbers NC_017416, NC_013130, NC_017401, www.ncbi.nlm.nih.gov). The reference N40 sequence for each gene is indicated with red asterisks. Variant nucleotides are indicated with colors. All three alignments contain a mix of two types of sequences, one identical to strain N40 reference sequence and a variant sequence identical to strain B31 reference sequence. Assemblies were performed in Geneious v 10.0.9 (www.geneious.com).

Next, we examined the assembled sequences of 16S rRNA-23S rRNA spacer region, and *ospC* and *dbpA* to determine the genotype of the infecting strains. Quality filtered reads were mapped to reference sequences from multiple *B. burgdorferi* s.s*.* strains (B31, N40, JD1, ZS7, WI39, and 297). Infecting strains were correctly identified as N40 in mouse 1, B31 in mouse 2 and tick 1, and a mix of both N40 and B31 strains present in mouse 3 (Fig. 2).

### Genotyping in ticks

To demonstrate the utility of TBDCapSeq for studies of ticks and detection of agents other than *B. burgdorferi* s.s., we selected nucleic acids from 18 individual ticks and two tick pools for TBDCapSeq analysis. These samples were all previously examined for the presence of tick-borne pathogens by UHTS or multiplex qPCR^37,38^. We selected 10 adult and 5 *I. scapularis* nymphs with infections with either one, two, or three agents. We also examined three individual *D. variabilis*, and two pools of *A. americanum* (pool 1, adults, N = 12; pool 2, nymphs, N = 11). The resulting Illumina reads were put through our regular bioinformatics pipeline, consisting of read filtration, contig assembly and homology searches through Blast. The TBDCapSeq results were 100% congruent with results obtained previously with UHTS or qPCR (Supplementary Table S1). We anticipated that the enrichment for pathogen-specific reads may not be as substantial compared to our experiments with mouse tissues, as the tick TNA had been subjected to > 3 freeze/thaw cycles prior to TBDCapSeq analysis. Nonetheless, in comparison to the previously obtained UHTS data, the fold enrichment of the sequencing reads with TBDCapSeq ranged from 20-fold to 74-fold for the five *I. scapularis* pathogens. We were also able to generate extended contigs that facilitated genomic analyses, including complete genome sequences from two tick samples positive for Powassan virus lineage II, and a 22 Kb *B. miyamotoi* chromosome contig from a *B. miyamotoi*-infected tick.

The presence of probes targeting rRNA genes of known pathogens also enabled detection of other closely related species. The rRNA probes for rickettsial pathogens resulted in the detection of the endosymbiont *Rickettsia buchneri* in 12 out of 15 *I. scapularis* samples, *Rickettsia amblyommatis* in both *A. americanum* pools, and *R. monacensis* in one of the two *D. variabilis* ticks. Ribosomal RNA probes also facilitated the detection of a novel *Babesia* species in one of the *A. americanum* pools. Analysis of assembled 18S rRNA and 28S rRNA contigs revealed that these sequences were most closely related to an unclassified *Babesia* species detected in white tailed deer from Texas (accession number HQ264120)^39^.

In some instances, highly homologous rRNA sequences also complicated species analysis. Initial BlastN analyses of sequences obtained from *Borrelia*-positive ticks identified 16S and 23S rRNA sequences as both B. burgdorferi s.s. and *B. miyamotoi*. Similar confounds occurred with *Babesia*-positive ticks, resulting with concurrent BlastN results to both of *B. microti* and *B. odocoilei* ribosomal genes. This applied only to sequences originating from portions of rRNA genes with high homology across species. To definitively delineate the species, examination of non-rRNA genes always resulted in the correct species identification.

### Pathogen detection in blood

We sought to determine the performance of TBDCapSeq on pathogens present in human blood samples. We first established the limits of detection of TBDCapSeq for *B. burgdorferi* s.s. and *B. microti* relative to qPCR, by quantifying and then serially diluting each pathogen in sterile human blood. For both agents, > 90% of every complete genomic segment (chromosome or plasmid) was recovered at parasitic/bacterial loads of approximately 50,000 genomic copies. In samples with the pathogen concentration reduced to approximately 1000 genomic copies, we still recovered > 25% of every segment from either agent.

*B. microti* samples 809-7 (Ct 38.1) and 813-8 (Ct 38.86) were the lowest dilutions tested on TBDCapSeq that were also positive by qPCR for *B. microti* (Supplementary Table S2). Both samples were estimated to contain 1–10 genomes of *B. microti.* We recovered > 1 Kb of sequence from each of the four *B. microti* chromosomes with TBDCapSeq from these samples. We also examined one subsequent dilution, 809-8, that was negative by qPCR, but were unable to identify unique *B. microti* reads in this sample.

*B. burgdorferi* s.s. sample LYM-846 (*ospA* Ct 36.65, *flaB* Ct 39.84) was the lowest qPCR positive sample tested and TBDCapSeq generated reads within in all major segments of the genome. The next dilution, LYM-847, was negative with both qPCR assays, but we were able to map 21 reads to cp32, lp17 and lp36 plasmids (Table 5).

**Table 5:** Comparison of the TBDCapSeq performance to qPCR on serially diluted *B. burgdorferi* N40 strain.

	LYM-841		LYM-842		LYM-843		LYM-844		LYM-845		LYM-846		LYM-847		LYM-849		LYM-850		Extraction control	
	*flaB* qPCR Ct	*ospA* qPCR Ct	*flaB* qPCR Ct	*ospA* qPCR Ct	*flaB* qPCR Ct	*ospA* qPCR Ct	*flaB* qPCR Ct	*ospA* qPCR Ct	*flaB* qPCR Ct	*ospA* qPCR Ct	*flaB* qPCR Ct	*ospA* qPCR Ct	*flaB* qPCR Ct	ospA qPCR Ct	flaB qPCR Ct	*ospA* qPCR Ct	*flaB* qPCR Ct	*ospA* qPCR Ct	*flaB* qPCR Ct	*ospA* qPCR Ct
	23.64	20.4	28.58	24.66	32.65	29.48	33.95	31.51	36.54	33.4	39.84	36.65	NA	NA	NA	NA	NA	NA	NA	NA
Raw Reads	35,063,602		22,486,207		16,710,629		23,554,898		19,813,832		18,287,462		17,456,214		19,060,206		18,835,547		18,978,900	
Filtered reads	27,287,508		16,310,431		11,124,968		16,189,953		13,617,696		12,295,407		10,615,240		13,187,179		12,985,727		13,215,357	
Reads mapped to B31	11,268,361		1,001,704		57,908		10,826		4954		491		73		35*		45*		32*	
% of reads mapped to B31	41.382		6.153		0.522		0.067		0.036		0.004		0.001		0		0		0	
Genomic Segment	Mapped reads (% coverage)																			
cp32-1	44,225 (94.41)		4449 (91.58)		318 (71.82)		51 (26.73)		29 (18.75)		1 (0.46)		2 (0.46)		0 (0)		0 (0)		0 (0)	
cp32-3	63,853 (97.82)		6482 (96.34)		399 (74.48)		75 (26.37)		42 (19.53)		7 (1.86)		1 (.047)		0 (0)		0 (0)		0 (0)	
cp-32-4	75,778 (95.07)		7811 (93.78)		546 (73.9)		99 (30.35)		81 (19.50)		3 (1.41)		1 (0.47)		0 (0)		0 (0)		0 (0)	
cp32-6	106,855 (90.73)		10,875 (88.53)		771 (75.0)		119 (32.55)		63 (20.16)		4 (1.42)		0		0 (0)		0 (0)		0 (0)	
cp32-7	43,573 (94.3)		4750 (92.13)		311 (68.99)		71 (26.35)		34 (14.34)		1 (1.38)		0		0 (0)		0 (0)		0 (0)	
cp32-8	43,055 (96.57)		4351 (94.15)		371 (72.19)		48 (27.67)		34 (17.30)		2 (0.92)		3 (0.46)		0 (0)		0 (0)		0 (0)	
cp32-9	76,973 (904.82)		7672 (93.71)		632 (73.78)		82 (28.73)		56 (19.38)		5 (0.93)		0		0 (0)		0 (0)		0 (0)	
lp21	3,980 (15.03)		356 (13.76)		12 (9.94)		0		0		0 (0)		0 (0)		0 (0)		0 (0)		0 (0)	
lp56	153,761 (69.57)		14,724 (67.47)		1030 (52.87)		191 (21.47)		97 (13.83)		5 (1.20)		0		0 (0)		0 (0)		0 (0)	
lp5	10,735 (45.83)		729 (44.17)		79 (32.48)		8 (13.01)		9 (10.35)		0 (0)		0 (0)		0 (0)		0 (0)		0 (0)	
CHR	7,521,512 (99.23)		677,488 (99.12)		38,572 (60.80)		7251 (15.61)		3171 (8.13)		330 (0.90)		52 (0.16)		35 (0.04)		45 (0.04)		31 (0.04)	
lp17	720,149 (99.4)		56,660 (99.31)		3196 (95.81)		662 (60.04)		383 (39.14)		38 (4.49)		3 (0.84)		0 (0)		0 (0)		0 (0)	
lp25	340,478 (100)		26,586 (99.8)		1584 (74.39)		244 (21.15)		185 (16.74)		12 (1.15)		0 (0)		0 (0)		0 (0)		0 (0)	
lp28-1	8018 (10.46)		534 (8.04)		45 (3.04)		8 (0.83)		0 (0)		1 (6.79)		0 (0)		0 (0)		0 (0)		0 (0)	
lp28-2	505,520 (99.72)		42,815 (99.64)		2115 (72.49)		246 (16.71)		192 (17.80)		33 (3.30)		0 (0)		0 (0)		0 (0)		0 (0)	
lp28-3	176,090 (86.8)		14,477 (85.84)		615 (45.17)		280 (24.52)		27 (5.73)		34 (2.85)		0 (0)		0 (0)		0 (0)		0 (0)	
lp28-4	220,059 (91.96)		17,158 (91.6)		1034 (64.07)		328 (23.01)		27 (5.88)		0 (0)		0 (0)		0 (0)		0 (0)		0 (0)	
lp36	82,139 (34.98)		6399 (32.37)		380 (20.54)		51 (5.78)		31 (2.45)		0 (0)		11 (0.71)		0 (0)		0 (0)		0 (0)	
lp38	5485 (9.98)		404 (9.47)		13 (6.48)		7 (2.53)		0		0 (0)		0 (0)		0 (0)		0 (0)		0 (0)	
lp54	724,635 (97.87)		62,690 (97.45)		3731 (76.25)		739 (26.62)		380 (17.78%)		15 (1.06)		0 (0)		0 (0)		0 (0)		1 (0.27)	
cp26	339,463 (98.74)		34,089 (97.41)		2128 (78.52)		266 (18.39)		113 (10.13)		0 (0)		0 (0)		0 (0)		0 (0)		0 (0)	
cp9	2025 (27.17)		205 (21.32)		26 (14.26)		0		0 (0)		0 (0)		0 (0)		0 (0)		0 (0)		0 (0)	

*All reads mapped to non-*Borrelia* 16S rRNA.

NA = no amplification.

### Clinical specimens

We examined a panel of 14 whole blood samples from patients diagnosed with acute babesiosis (Supplementary Table S3). For five patients, we also analyzed samples collected at a post-treatment follow up visit. The parasitic load was determined by qPCR. Ten samples had high parasitemia as determined by qPCR, with a Ct range of 17.84 to 23.41 (Corresponding to ~ 10^7^ to ~ 2.5 × 10^5^ copies of *coxA*). TBDCapSeq analysis of these samples resulted in a remarkable enrichment for *Babesia* reads. Between 81.2 and > 98.9% of all reads from these samples mapped to *B. microti*. Consequently, we were able to assemble the complete sequence (> 99.8% coverage) of the four *B. microti* chromosomes from each of these samples. Comparison of these assembled sequences to the *B. microti* reference strain RI revealed only a limited number of mostly synonymous nucleotide substitutions. Notable exceptions were several ORFs predominately on chromosome 4 that displayed a greater number of nucleotide variations when compared to the reference sequences. Among them were the Bmn 1-11 and Bmn 1-15 genes that encode putative immunogenic outer surface proteins (Supplementary Fig. S1).

Three samples with lower *Babesia* read counts by TBDCapSeq had low parasitemia as determined by qPCR (Cts 33.69 to 38.47). Five samples, all from follow up visits, were negative for *B. microti* by qPCR, but we obtained > 100 *Babesia* non rRNA reads from each sample with TBDCapSeq.

Next, we examined 18 blood samples from patients with an erythema migrans (EM) (N = 15) or acute babesiosis (N = 3) (Supplementary Table S4). All Lyme disease samples were negative by an *ospA* qPCR. The TBDCapSeq data from this pool were heavily biased towards babesiosis samples with high pathogen burden. The two babesiosis samples with high parasitemia (Ct < 18) accounted for > 87% of all reads on the flow cell, with > 97% of reads from each sample mapping to *B. microti*. In two of the EM samples, we were able to identify *B. burgdorferi* s.s. reads, although at low quantities. Sample LYM-904 had 60 non-ribosomal reads, all mapping to cp32 plasmids. LYM-912 had 57 reads, with the majority (N = 24) mapping to Cp32. Neither sample contained chromosomal reads outside of rRNA genes.

## Discussion

In this study, we demonstrated the utility of TBDCapSeq for simultaneous detection and genotyping of tick-borne disease agents in a wide range of sample matrices. Although our primary focus was on detection of *B. burgdorferi* s.s. and *B. microti*, in our experiments with field-collected ticks we demonstrated the utility of TBDCapSeq for simultanous detection and differentiation of other tick-borne agents. We found TBDCapSeq to be markedly superior in performance to UHTS, and in some instances, it exceeded the sensitivity of qPCR. This was a crucial finding, as assay sensitivity is one of the primary concerns with molecular assays targeting tick-borne agents. For pathogens such as *B. microti* or *A. phagocytophilum*, molecular detection in the acute stage can be straight forward^3^. However, the paucity of spirochetes in blood has proven to be a considerable challenge for molecular detection of *B. burgdorferi *sensu lato. Serology has been shown to be more useful in Lyme disease diagnosis, but currently employed serologic assays can also suffer from intrinsic limitations, including inadequate sensitivity and specificity for samples collected early in disease as well as subjectivity in data interpretation^11,40–42^. As a result, alternative platforms for diagnosis of acute Lyme disease have been pursued, including metabolomics, transcriptomics and modifications of PCR such as digital droplet PCR^43–45^. In specimens with active bacteremia, TBDCapSeq can address the sensitivity limitations of other molecular assays and provide a new approach for genomic and pathogenesis studies of *B. burgdorferi* s.s*.* and other tick-borne agents. The limit of detection of TBDCapSeq was at, or below, the detection limits of qPCR. This promising result can potentially be further magnified with modifications to sample preparations and sequencing protocols. For example, increasing the sample volume may partially offset the paucity of spirochetes in liquid specimens and enhance detection. In our experiments, we used nucleic acids extracted from only 200 μl of whole blood, in contrast to other molecular studies of *B. burgdorferi* s.s. that typically employ much greater sample volumes for spirochete detection (20 ml of whole blood or 1 ml of platelet-rich plasma)^44,46^. Increasing sequencing depth may also result in greater yield of pathogen-specific reads. In future tests, we will seek to further enhance assay performance in order to determine its utility on complex specimens that typically yield limited data with molecular assays.

Our strategy for identification and quantification of pathogen sequences consisted primarily of mapping sequencing reads directly to a reference genome. For *B. burgdorferi* s.s*.*, this approach could potentially underestimate the actual number of reads due to mismatches in polymorphic sequences. To account for strain differences, for several samples where the infecting strain was determined to be other than B31, we mapped the reads to other *B. burgdorferi* s.s*.* strains. We did not detect a significant difference in the number of *Borrelia* reads when mapping to these other strains, and occasionally, the output was lower due to the fact that for some strains the complete genome sequence has not yet been deposited in GenBank. We also observed that in all samples, because of high sequence homology, a small subset of ribosomal reads originating from the host (mouse, human or tick) or environmental bacteria mapped to conserved regions in rRNA genes of the reference pathogen genome. As a result, ribosomal reads were omitted from our analyses, particularly in samples with a low pathogen burden.

The combination of genome-level analysis with unparalleled detection capability offered by TBDCapSeq can have immense implications on studies of TBD. This assay could offer valuable new insights into our understanding of TBD by facilitating analysis of previously challenging specimens.

## Methods

### Probe design

TBDCapseq capture probes were designed to target the most common agents of TBD found in the US (Supplementary Table S5). A reference sequence for every genetic segment of each agent was used as template for probe design. To account for the high degree of heterogeneity in *B. burgdorferi* s.s. plasmid sequences we included three strains representing disparate OspC types (A, K and E). Probes were designed along the entire nucleotide sequence of every genomic segment, with a total of 106 plasmid and chromosomal sequences used in the design. The final set consisted of > 400,000 probes. Probes were manufactured by Roche Sequencing Solutions as previously described^24,25^.

### Samples

We analyzed 7 TBDCapSeq runs, designated as experiments 1–7. Tissue samples analyzed in experiments 1 and 2 were obtained from C3H mice infected by needle inoculation with 1 × 10^5^ of cultured infectious strains of *B. burgdorferi* s.s. (N40, B31, or both). Larval ticks were infected by feeding on the *B. burgdorferi* s.s*.*-infected mice and were then frozen. Mice were bred and maintained in the Tufts University Animal Facility. All experiments were performed following the guidelines of the American Veterinary Medical Association (AVMA) as well as the Guide for the Care and Use of Laboratory Animals of the National Institutes of Health. All procedures were performed with approval of the Tufts University Institutional Animal Care and Use Committee. Euthanasia was performed in accordance with guidelines provided by the AVMA and was approved by the Tufts University IACUC. All methods were in accordance with ARRIVE guidelines. For analyses of unfed nymphs and adult ticks in experiment 3, we used cDNA generated in previous tick studies^37,38^.

Whole blood samples were obtained from patients presenting with a tick-borne illness. Acute babesiosis cases with parasitemia by blood smear (*Babesia microti* confirmed by PCR), Lyme disease cases (serological CDC criteria or erythema migrans diagnosed by a physician) and controls (serology negative for Lyme disease) were enrolled at Stony Brook University Hospital (IRB#1210472). Additional whole blood specimens from patients diagnosed with early localized Lyme disease (all with erythema migrans) were obtained from Columbia University’s Lyme and Tick-Borne Diseases Research Center at Columbia University.

For sensitivity tests, we generated contrived whole blood samples by spiking-in quantified pathogens followed by serial dilutions. For tests with *B. burgdorferi* s.s*.*, we serially diluted a culture of an infectious N40 strain. For experiments with *B. microti*, we serially diluted *B. microti* from a pair of clinical whole blood samples. For both agents, samples were initially diluted 1:10 four times, followed by multiple subsequent 1:5 dilutions.

To estimate pathogen burden, we used 5 μl of template in quantitative PCR (qPCR) assays for *B. burgdorferi* s.s. *(ospA and flaB)* and *B. microti* (*coxA)*^37,47^. All qPCR assays were performed using the TaqMan universal PCR master mix (Applied Biosystems).

### Sample extractions

Nucleic acid extractions were performed using multiple methods. For extraction of mouse tissues, 1 ml phosphate buffered saline and 1 μl of Dx solution (Qiagen) was added to tissue fragments followed by addition of 1 mm glass beads and homogenization. Ten μl of proteinase K was added, incubated 65 °C for 30 min, followed by centrifugation of cellular debris. Total nucleic acid (TNA) from 250 μl of the supernatant from mouse tissue samples in experiments 1 and 2 (Table 1) were extracted using the Easy Mag extraction platform (Biomerieux) and eluted in 40 μl. TNA from all tick samples examined in experiment 3 were also extracted using the EasyMag. DNA from whole blood (experiments 4–7) was extracted using 200 μl of each sample with the QIAamp DNA Blood Mini Kit (Qiagen) and eluted in 50 μl of water.

To establish detection thresholds and determine the vigor of the workflow, we used pre-characterized tick-borne pathogen-free samples, including salmon sperm DNA, and whole blood specimens obtained from the Columbia University Pathology department.

### HTS sequencing and liquid capture methods

DNA concentrations were measured with the Qubit High Sensitivity Double-stranded DNA kit and Qubit 2.0 Fluorometer (Invitrogen). Dual indexed libraries were prepared with the Kapa Hyperplus kit (Roche) using 25–50 ng of input material and the recommended adaptor concentrations and cycling parameters. Amplified libraries were quantified on a TapeStation 4200 using the D1000 kit (Agilent Technologies). Measured concentrations were used to pool libraries at 150 ng per library. After quantification on the TapeStation 4200, 1 μg of the pool was mixed with 5 μg of COT Human DNA (Thermo Fisher Scientific) and 2000 pmol of Blocking Oligo pool (Roche). The mixture was fully dehydrated at 60 °C in a vacuum centrifuge. The dried pool was resuspended in 7.5 μl Hybridization Buffer and 3 μl Hybridization Component A (Roche) to a volume of 10.5 μl and heated at 95 °C for 5 min before the addition of 4.5 μl of custom biotinylated TBD SeqCap EZ Probe pool (Roche). The mixture was again heated at 95 °C for 5 min before being incubated at 47 °C for 16–20 h. After incubation, the probes were pulled down using magnetic streptavidin SeqCap Capture beads (Roche) and washed with buffers of decreasing stringency (Wash Buffers I, II, III, and IV, Roche). The probe-bound DNA was eluted in water and amplified by 16 cycles of PCR using Illumina universal primers (Kapa HiFi HotStart Ready Mix, Roche) using Illumina Universal Primers. Finally, the amplified pool was quantified (Agilent Tapestation) and sequenced on an Illumina NextSeq2000 platform that generated 150 bp long single end reads.

### Bioinformatic analyses

The fastq files were adapter trimmed using Cutadapt program (v 3.0)^48^. Adaptor trimming was followed by generation of quality reports using FastQC software, (v 0.11.5)^49^ which were used to determine trimming and filtering criteria based on the average quality scores, read length, homopolymeric reads, nucleotide bias and quality scores at the ends of the reads. The reads were quality filtered and end-trimmed with PRINSEQ software (v 0.20.3)^50^. To determine the abundance of *B. burgdorferi* s.s*.* reads, a database was created by downloading the reference sequences of the 21 plasmids and the linear chromosome of the B31 strain from the NCBI RefSeq database. The reference sequences were used to create a Bowtie2 index and the quality filtered reads were mapped to the database using Bowtie2 mapper (v 2.2.9)^51^. The bam files containing mapped reads were parsed using a set of custom scripts that utilize the SAMtools and BEDTools for extracting depth and breadth of coverage for each genomic and plasmid sequence. A similar process was followed for *B. microti* strain RI by downloading the 4 chromosomes, mitochondrion and apicoplast sequences from NCBI RefSeq database. Pathogen DNA free controls were used to discern the quantity of mis-assigned reads for each run.

For experiment 3 only, we followed our standard pipeline for agent identification^24,38^. Host reads were removed by mapping quality filtered reads against tick reference database using Bowtie2 mapper. The host-subtracted reads were de-novo assembled using MIRA (4.0) assembler^52^. Contigs and unique singletons were subjected to homology search using Megablast against the complete GenBank nucleotide database. Sequences that showed poor or no homology at the nucleotide level were screened with BLASTX against the viral GenBank protein database. The blast reports were annotated with the taxonomic information from NCBI taxonomy database and the reports were used to identify accession numbers for candidate genomes for mapping the reads and determining the genomic coverage and depth.

## Supplementary Information

Supplementary Information.

## Data Availability

All high-throughput sequencing data has been deposited in the Sequence Read Archive under BioProject ID PRJNA723600. *Babesia* sequences were deposited in GenBank under accession numbers MW665112-MW665119.
