# Supplementary material for: Development of a capture sequencing assay for enhanced detection and genotyping of tick-borne pathogens
Source: Sci Rep. 2021 Jun 11;11:12384. doi: 10.1038/s41598-021-91956-z (PMC8196166; doi:10.1038/s41598-021-91956-z)
Supplement: Supplementary file 1 — Supplementary Information. [file 41598_2021_91956_MOESM1_ESM.pdf]

SUPPLEMENTARY INFORMATION

Development of a capture sequencing assay for enhanced detection and genotyping of tick-borne pathogens.

Komal Jain[1], Teresa Tagliafierro[1], Adriana Marques[2], Santiago Sanchez-Vicente[1], Alper Gokden[1], Brian Fallon[3], Nischay Mishra[1,4], Thomas Briese[1], Vishal Kapoor[1], Stephen Sameroff[1], Cheng Guo[1], Luis A. Marcos[5], Linden Hu[6], W. Ian Lipkin[1,4], Rafal Tokarz[1,4]

[1]Center for Infection and Immunity, Mailman School of Public Health, Columbia University, New York, NY

[2]Laboratory of Clinical Immunology and Microbiology, National Institute of Allergy and Infectious Diseases, National Institutes of Health, Bethesda, Maryland.

[3]Department of Psychiatry, Columbia University, New York, NY

[4]Department of Epidemiology, Mailman School of Public Health, Columbia University, New York, NY

[5]Department of Medicine (Division of Infectious Diseases) and Department of Microbiology and Immunology, State University of New York at Stony Brook, Stony Brook, NY

[6]Department of Molecular Biology and Microbiology, Tufts University, Boston, Massachusetts, USA.

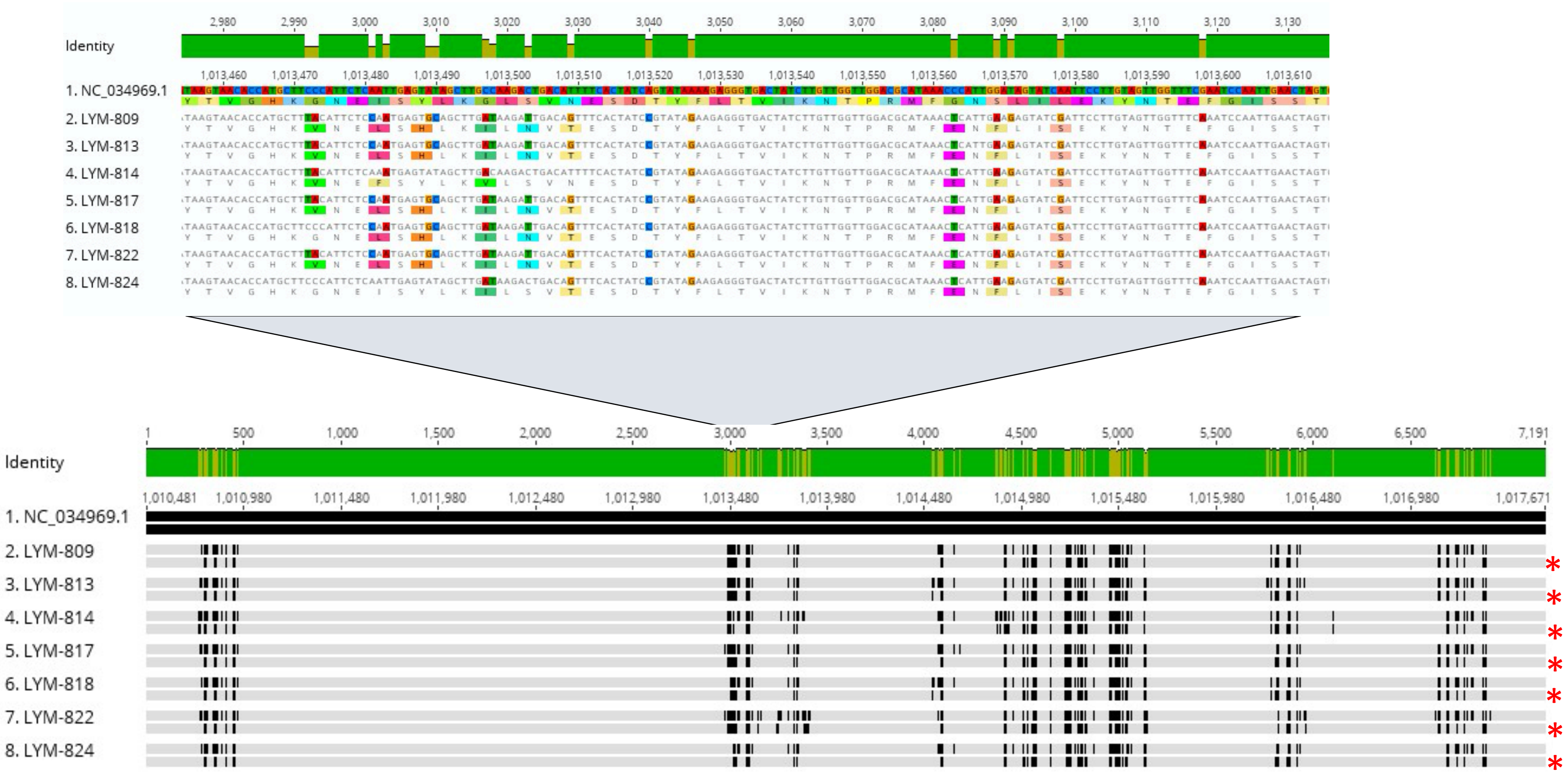

Supplementary Fig. S1. Alignment of *B. microti bmn 1-15* sequences recovered by TBDCapSeq from clinical samples. Shown are seven sequences (LYM-809, LYM-813, LYM-814, LYM-817, LYM-818, LYM-822 and LYM-824) aligned to a reference sequence NC_034969 (www.ncbi.nlm.nih.gov). The bottom alignment consists of the complete open reading frame (position 1-7191), with nucleotide sequence (top) and their amino acid translations (bottom-with asterisk). Discrepancies to the reference sequence are highlighted in black. The top alignment displays an enlarged portion of the region between nt 2650 and 3070 with variant nucleotides and amino acids displayed in color. Assemblies were performed in Geneious v 10.0.9 (www.geneious.com).

Supplementary Table S1. Comparison of TBDCapSeq vs qPCR and UHTS for surveillance of infected ticks.

		**Sample ID**	NYT-149	NYT-150	NYT-209	NYT-219	NYT-224	NYT-243	NYT-245	NYT-246	NYT-285	NYT-328
		**Sample type**	*Ixodes scapularis* nymph	*Ixodes scapularis* nymph	*Ixodes scapularis* nymph	*Ixodes scapularis* nymph	*Ixodes scapularis* nymph	*Ixodes scapularis* adult male	*Ixodes scapularis* adult male	*Ixodes scapularis* adult male	*Ixodes scapularis* adult female	*Ixodes scapularis* adult male
**Unbiased Sequencing**		**Total raw reads**	ND	ND	ND	ND	ND	ND	ND	ND	ND	ND
		**# Reads after Filtration**	ND	ND	ND	ND	ND	ND	ND	ND	ND	ND
	**Reads per million filtered reads**	***B. burgdorferi***	ND	ND	ND	ND	ND	ND	ND	ND	ND	ND
		B. miyamotoi	ND	ND	ND	ND	ND	ND	ND	ND	ND	ND
		**Powassan virus**	ND	ND	ND	ND	ND	ND	ND	ND	ND	ND
		A. phagocytophilum	ND	ND	ND	ND	ND	ND	ND	ND	ND	ND
		B. microti	ND	ND	ND	ND	ND	ND	ND	ND	ND	ND
		B. odocoilei	ND	ND	ND	ND	ND	ND	ND	ND	ND	ND
		Rickettsia	ND	ND	ND	ND	ND	ND	ND	ND	ND	ND
**Capture Sequencing (fold enrichment relative to UHTS)**		**Total raw reads**	3,374,160	3,365,391	4,333,176	31,183,867	8,107,305	3,440,268	3,945,563	1,017,337	5,930,917	1,638,322
		**# Reads after Filtration**	2,967,017	2,999,786	3,934,890	28,087,631	7,317,302	3,165,477	3,540,779	904,830	5,432,860	1,470,345
	**Reads per million filtered reads (fold increase)**	***B. burgdorferi***	-	138,174	4,891	17,796	64,052	406,056	-	-	114,839	-
		B. miyamotoi	-		546,825	-	-	-	-	-	-	-
		**Powassan virus**	-	-	-	-	-	-	654,136	-	-	-
		A. phagocytophilum	-	-	-	-	161,478	-	-	-	-	69,578
		B. microti	156,421	-	-	783,424	-	-	-	-	-	-
		B. odocoilei	-	-	-	-	-	-	-	-	-	-
		Rickettsia	22,737	15,717	2,354	488	11,930	-	-	-	41,949	9,009
**RT-PCR results (Ct)**		***B. burgdorferi***	-	Pos (29.64)	Pos (29.58)	Pos (30.98)	Pos (29.00)	Pos (24.73)	-	-	Pos (25.64)	-
		B. miyamotoi	-	-	Pos (26.33)	-	-	-	-	-	-	-
		**Powassan virus**	-	-	-	-	-	-	Pos (24.27)	-	-	-
		A. phagocytophilum	-	-	-	-	Pos (18.19)	-	-	-	Pos (30.09)	Pos (19.77)
		B. microti	Pos (28.45)	-	-	Pos (25.64)	-	-	-	-	-	-

ND - not done

Supplementary Table S1. Comparison of TBDCapSeq vs qPCR and UHTS for surveillance of infected ticks (continuation).

		Sample ID	NYT-380	NYT-381	NYT-382	NYT-435	NYT-436	RTS-619	RTS-689	RTS-694	RTS-695	RTS-706
		Sample type	*Dermacentor variabilis adult female*	*Dermacentor variabilis adult female*	*Dermacentor variabilis adult female*	*Amblyomma americanum adult pool*	*Amblyomma americanum nymph pool*	*Ixodes scapularis* adult female	*Ixodes scapularis* adult male	*Ixodes scapularis* adult female	*Ixodes scapularis* adult female	*Ixodes scapularis* adult female
**Unbiased Sequencing**		**Total raw reads**	ND	ND	ND	ND	ND	16,825,510	8,042,901	5,191,483	6,253,426	8,217,771
		**# Reads after Filtration**	ND	ND	ND	ND	ND	15,427,713	7,485,930	4,851,515	5,889,894	7,705,660
	**Reads per million filtered reads**	***B. burgdorferi***	ND	ND	ND	ND	ND	341	103	0	114	-
		B. miyamotoi	ND	ND	ND	ND	ND	-	-	4,071	-	-
		**Powassan virus**	ND	ND	ND	ND	ND	13,584	-	0	-	-
		A. phagocytophilum	ND	ND	ND	ND	ND	91	-	420	1,688	-
		B. microti	ND	ND	ND	ND	ND	-	-	0	2,914	-
		B. odocoilei	ND	ND	ND	ND	ND	410	-	0	0	1,386
		Rickettsia	ND	ND	ND	ND	ND	21	4,796	1,145	3,247	1,155
**Capture Sequencing (fold enrichment relative to UHTS)**		**Total raw reads**	1,778,385	1,607,649	1,571,138	7,131,575	3,312,333	7,730,715	22,780,772	10,430,420	25,182,055	11,802,060
		**# Reads after Filtration**	1,614,403	1,458,241	1,416,267	6,622,463	3,036,827	7,038,868	21,211,157	9,751,093	23,243,365	10,701,822
	**Reads per million filtered reads (fold increase)**	***B. burgdorferi***	-	-	-	-	-	25,179 (73.8)	3,901 (37.9)	-	4,514 (39.7)	-
		B. miyamotoi	-	-	-	-	-	-	-	329,154 (80.8)	-	-
		**Powassan virus**	-	-	-	-	-	708,945 (52.2)	-	-	-	-
		A. phagocytophilum	-	-	-	-	-	5,389 (59.1)	-	22,200 (52.8)	34,622 (20.5)	-
		B. microti	-	-	-	-	-	-	-	-	74,187 (25.5)	-
		B. odocoilei	-	-	-	-	-	3,950 (9.6)	-	-	-	7,492 (5.4)
		Rickettsia	-	201	-	11,475	2,625	815 (39.1)	84,865 (17.7)	42,869 (37.4)	68,643 (21.1)	40,359 (34.9)
**RT-PCR results (Ct)**		***B. burgdorferi***	-	-	-	-	-	Pos (31.06)	-	-	-	-
		B. miyamotoi	-	-	-	-	-	-	-	Pos (26.02)	-	-
		**Powassan virus**	-	-	-	-	-	Pos (26.57)	-	-	-	-
		A. phagocytophilum	-	-	-	-	-	Pos (26.2)	-	Pos (18.98)	Pos (15.68)	-
		B. microti	-	-	-	-	-	-	-		Pos (25.08)	-

ND - not done

Supplementary Table S2. Serial dilution experiments with *B. microti*

Sample	Number rawfastq reads	Number input reads	Number of unique mapped reads (% mapped)	Number mapped reads	Reference Name (reference length)	Length Genome Recovered	% genome recovered	qPCR Ct
LYM-809-1	63,092,901	54,926,856	43,416,904 (79.05%)	8,905,599	NC_027205 (1,304,281)	1,304,181	99.99%	23.01
				10,107,952	NC_027206 (1,508,385)	1,508,133	99.98%	
				11,973,552	NC_027207 (1,763,809)	1,763,809	99.85%	
				12,320,825	NC_034969 (1,816,206)	1,815,055	99.94%	
LYM-809-2	25,466,254	21,095,128	8,885,664 (42.12%)	1,825,592	NC_027205 (1,304,281)	1,299,343	99.62%	NOT DONE
				2,071,503	NC_027206 (1,508,385)	1,499,825	99.43%	
				2,476,634	NC_027207 (1,763,809)	1,754,806	99.34%	
				2,482,592	NC_034969 (1,816,206)	1,806,164	99.45%	
LYM-809-3	14,840,955	11,710,156	159,414 (1.36%)	29,181	NC_027205 (1,304,281)	156,398	11.99%	NOT DONE
				35,480	NC_027206 (1,508,385)	177,153	11.75%	
				51,580	NC_027207 (1,763,809)	211,641	11.98%	
				42,699	NC_034969 (1,816,206)	218,186	12.01%	
LYM-809-4	13,104,169	10,440,591	95,345 (0.10%)	14,985	NC_027205 (1,304,281)	84,571	6.48%	NOT DONE
				20,721	NC_027206 (1,508,385)	112,116	7.43%	
				36,236	NC_027207 (1,763,809)	144,527	8.18%	
				23,114	NC_034969 (1,816,206)	124,060	6.83%	
LYM-809-5	13,744,779	10,721,711	32,409 (0.30%)	4,100	NC_027205 (1,304,281)	24,230	1.86%	NOT DONE
				5,507	NC_027206 (1,508,385)	30,352	2.01%	
				17,124	NC_027207 (1,763,809)	36,397	2.06%	
				5,611	NC_034969 (1,816,206)	30,767	1.69%	
LYM-809-6	12,385,307	9,843,502	19,768 (0.20%)	2,041	NC_027205 (1,304,281)	11,732	0.90%	36.85
				2,568	NC_027206 (1,508,385)	14,086	0.93%	
				13,095	NC_027207 (1,763,809)	18,683	1.06%	
				1,950	NC_034969 (1,816,206)	11,002	0.61%	
LYM-809-7	11,914,908	9,451,396	12,554 (0.13%)	424	NC_027205 (1,304,281)	2,464	0.19%	38.1
				480	NC_027206 (1,508,385)	3,709	0.25%	
				11,356	NC_027207 (1,763,809)	7,882	0.45%	
				294	NC_034969 (1,816,206)	1,963	0.11%	
LYM-809-8	12,914,339	10,208,926	10,400 (0.10%)	2	NC_027205 (1,304,281)	284	0.02%	NEGATIVE
				45	NC_027206 (1,508,385)	335	0.02%	
				10,336	NC_027207 (1,763,809)	5,341	0.30%	
				17	NC_034969 (1,816,206)	142	0.01%	
LYM-813-1	113,600,674	100,768,336	89,237,158 (88.56%)	18,213,981	NC_027205 (1,304,281)	1,304,211	100.00%	23.16
				20,815,876	NC_027206 (1,508,385)	1,508,147	99.98%	
				24,626,856	NC_027207 (1,763,809)	1,763,831	99.85%	
				25,269,761	NC_034969 (1,816,206)	1,815,078	99.94%	
LYM-813-2	39,064,619	34,023,940	23,942,511 (70.37%)	4,888,370	NC_027205 (1,304,281)	1,304,183	99.99%	25.58
				5,571,830	NC_027206 (1,508,385)	1,508,117	99.98%	
				6,648,132	NC_027207 (1,763,809)	1,763,651	99.84%	
				6,768,021	NC_034969 (1,816,206)	1,814,974	99.93%	
LYM-813-3	16,704,258	13,566,946	2,344,511 (17.28%)	479,935	NC_027205 (1,304,281)	1,069,353	81.99%	27.23
				542,730	NC_027206 (1,508,385)	1,217,288	80.70%	
				651,796	NC_027207 (1,763,809)	1,433,680	81.16%	
				661,831	NC_034969 (1,816,206)	1,473,271	81.12%	
LYM-813-4	19,057,117	15,062,202	501,790 (3.33%)	103,202	NC_027205 (1,304,281)	407,930	31.28%	30.15
				111,387	NC_027206 (1,508,385)	443,655	29.41%	
				146,826	NC_027207 (1,763,809)	533,512	30.20%	
				138,285	NC_034969 (1,816,206)	551,416	30.36%	
LYM-813-5	17,351,276	13,600,010	87,680 (0.65%)	14,408	NC_027205 (1,304,281)	71,234	5.46%	31.28
				17,220	NC_027206 (1,508,385)	83,605	5.54%	
				32,748	NC_027207 (1,763,809)	94,032	5.32%	
				22,924	NC_034969 (1,816,206)	108,424	5.97%	
LYM-813-6	18,969,775	14,973,275	40,258 (0.27%)	4,805	NC_027205 (1,304,281)	29,914	2.29%	33.42
				4,457	NC_027206 (1,508,385)	22,397	1.49%	
				24,176	NC_027207 (1,763,809)	42,727	2.42%	
				6,647	NC_034969 (1,816,206)	33,510	1.85%	
LYM-813-7	16,602,699	13,093,379	19,988 (0.15%)	1,152	NC_027205 (1,304,281)	7,593	0.58%	37.12
				1,111	NC_027206 (1,508,385)	6,209	0.41%	
				16,044	NC_027207 (1,763,809)	11,027	0.62%	
				1,673	NC_034969 (1,816,206)	9,280	0.51%	
LYM-813-8	15,389,067	11,912,012	15,521 (0.13%)	197	NC_027205 (1,304,281)	1,477	0.11%	38.86
				229	NC_027206 (1,508,385)	1,117	0.07%	
				14,553	NC_027207 (1,763,809)	6,722	0.38%	
				542	NC_034969 (1,816,206)	2,881	0.16%	
Salmon Sperm DNA	21,881,921	10,261,592	36,536 (0.36%)	4	NC_027205 (1,304,281)	542	0.04%	NOT DONE
				5	NC_027206 (1,508,385)	679	0.05%	
				36,312*	NC_027207 (1,763,809)	7,072	0.40%	
				122	NC_034969 (1,816,206)	1096	0.06%	
Extraction Control	14,326,099	11,332,867	13,058 (0.12%)	62	NC_027205 (1,304,281)	426	0.03%	NOT DONE
				41	NC_027206 (1,508,385)	142	0.01%	
				12,955*	NC_027207 (1,763,809)	3,959	0.22%	
				0	NC_034969 (1,816,206)	0	0.00%	

*reads mapped to host (human) 18S rRNA and 28S rRNA

Supplementary Table S3. TBDCapSeq analysis of whole blood samples from patients with babesiosis.

Sample Name	Sample ID	Ct	Percentage of mapped reads to *B. microti*	Number of mapped reads	Ref. Name	Genome recovered (%)
HEALTHY CONTROL	Neg Control			0	NC_027205 (chromosome I)	0.00%
	Neg Control	ND	0.231	0	NC_027206 (chromosome II)	0.00%
	Neg Control			1251*	NC_027207 (chromosome III)	0.25%
	Neg Control			0	NC_034969 (chromosome IV)	0.00%
PATIENT 1	LYM-822			335,619	NC_027205 (chromosome I)	99.99%
	LYM-822	23.41	81.179	382,555	NC_027206 (chromosome II)	99.97%
	LYM-822			451,283	NC_027207 (chromosome III)	99.85%
	LYM-822			463,049	NC_034969 (chromosome IV)	99.93%
PATIENT 1	LYM-819			64	NC_027205 (chromosome I)	0.49%
FOLLOW UP VISIT	LYM-819	NA	0.322	87	NC_027206 (chromosome II)	0.53%
	LYM-819			1,265	NC_027207 (chromosome III)	0.89%
	LYM-819			161	NC_034969 (chromosome IV)	0.68%
PATIENT 2	LYM-813			12,200,073	NC_027205 (chromosome I)	100.00%
	LYM-813	17.84	98.931	13,853,115	NC_027206 (chromosome II)	99.98%
	LYM-813			16,391,885	NC_027207 (chromosome III)	99.85%
	LYM-813			16,895,013	NC_034969 (chromosome IV)	99.94%
PATIENT 2	LYM-826			137	NC_027205 (chromosome I)	0.92%
FOLLOW UP VISIT	LYM-826	NA	0.19	161	NC_027206 (chromosome II)	0.83%
	LYM-826			1,141	NC_027207 (chromosome III)	0.91%
	LYM-826_			172	NC_034969 (chromosome IV)	0.80%
PATIENT 3	LYM-811			149	NC_027205 (chromosome I)	0.96%
	LYM-811	35.15	0.481	124	NC_027206 (chromosome II)	0.72%
	LYM-811			1,815	NC_027207 (chromosome III)	1.26%
	LYM-811			193	NC_034969 (chromosome IV)	1.02%
PATIENT 3	LYM-810			113	NC_027205 (chromosome I)	0.74%
FOLLOW UP VISIT	LYM-810	NA	0.512	132	NC_027206 (chromosome II)	0.79%
	LYM-810			1,645	NC_027207 (chromosome III)	1.34%
	LYM-810			226	NC_034969 (chromosome IV)	0.94%
PATIENT 4	LYM-809			5,186,474	NC_027205 (chromosome I)	100.00%
	LYM-809	19.32	97.558	5,867,246	NC_027206 (chromosome II)	99.98%
	LYM-809			6,975,669	NC_027207 (chromosome III)	99.86%
	LYM-809			7,174,369	NC_034969 (chromosome IV)	99.93%
PATIENT 5	LYM-813			12,200,073	NC_027205 (chromosome I)	100.00%
	LYM-813	17.84	98.931	13,853,115	NC_027206 (chromosome II)	99.98%
	LYM-813			16,391,885	NC_027207 (chromosome III)	99.85%
	LYM-813			16,895,013	NC_034969 (chromosome IV)	99.94%
PATIENT 6	LYM-814			403,215	NC_027205 (chromosome I)	99.99%
	LYM-814	22.88	84.721	458,123	NC_027206 (chromosome II)	99.98%
	LYM-814			545,801	NC_027207 (chromosome III)	99.84%
	LYM-814			561,845	NC_034969 (chromosome IV)	99.92%
PATIENT 7	LYM-815			627,894	NC_027205 (chromosome I)	99.99%
	LYM-815	21.72	87.493	715,626	NC_027206 (chromosome II)	99.98%
	LYM-815			850,275	NC_027207 (chromosome III)	99.85%
	LYM-815			876,962	NC_034969 (chromosome IV)	99.93%
PATIENT 8	LYM-816			938,495	NC_027205 (chromosome I)	99.99%
	LYM-816	22.67	91.585	1,069,502	NC_027206 (chromosome II)	99.98%
	LYM-816			1,281,236	NC_027207 (chromosome III)	99.85%
	LYM-816			1,305,914	NC_034969 (chromosome IV)	99.93%
PATIENT 9	LYM-817			4,342,838	NC_027205 (chromosome I)	100.00%
	LYM-817	ND	96.571	4,971,311	NC_027206 (chromosome II)	99.98%
	LYM-817			5,880,114	NC_027207 (chromosome III)	99.86%
	LYM-817			6,087,173	NC_034969 (chromosome IV)	99.94%
PATIENT 10	LYM-818			15,581,725	NC_027205 (chromosome I)	100.00%
	LYM-818	18.65	98.745	17,486,839	NC_027206 (chromosome II)	99.99%
	LYM-818			20,874,686	NC_027207 (chromosome III)	99.86%
	LYM-818			21,331,613	NC_034969 (chromosome IV)	99.94%
PATIENT 11	LYM-821			250	NC_027205 (chromosome I)	1.56%
	LYM-821	38.47	0.487	273	NC_027206 (chromosome II)	1.40%
	LYM-821			1,612	NC_027207 (chromosome III)	1.53%
	LYM-821			288	NC_034969 (chromosome IV)	1.41%
PATIENT 12	LYM-824			10,649,119	NC_027205 (chromosome I)	100.00%
	LYM-824	18.07	98.496	12,176,315	NC_027206 (chromosome II)	99.99%
	LYM-824			14,377,969	NC_027207 (chromosome III)	99.86%
	LYM-824			14,820,064	NC_034969 (chromosome IV)	99.93%
PATIENT 13	LYM-825			17	NC_027205 (chromosome I)	0.10%
FOLLOW UP VISIT	LYM-825	NA	0.471	20	NC_027206 (chromosome II)	0.08%
	LYM-825			1,834	NC_027207 (chromosome III)	0.30%
	LYM-825				NC_034969 (chromosome IV)	
PATIENT 14	LYM-827			64	NC_027205 (chromosome I)	0.41%
	LYM-827	33.69	0.238	93	NC_027206 (chromosome II)	0.51%
	LYM-827			1,177	NC_027207 (chromosome III)	0.73%
	LYM-827			143	NC_034969 (chromosome IV)	0.45%
PATIENT 15	LYM-828			32,224,882	NC_027205 (chromosome I)	100.00%
	LYM-828	19.04	97.747	36,546,749	NC_027206 (chromosome II)	99.98%
	LYM-828			43,406,519	NC_027207 (chromosome III)	99.87%
	LYM-828			45,788,830	NC_034969 (chromosome IV)	99.95%
PATIENT 16	LYM-820			30	NC_027205 (chromosome I)	0.20%
FOLLOW UP VISIT	LYM-820	NA	0.338	33	NC_027206 (chromosome II)	0.16%
	LYM-820			1,315	NC_027207 (chromosome III)	0.44%
	LYM-820			38	NC_034969 (chromosome IV)	0.19%
Salmon sperm	Negative control			0	NC_027205 (chromosome I)	0.00%
Salmon sperm	Negative control	ND	0.881	0	NC_027206 (chromosome II)	0.00%
Salmon sperm	Negative control			1883*	NC_027207 (chromosome III)	0.25%
Salmon sperm	Negative control			8	NC_034969 (chromosome IV)	0.01%

*all reads mapped to host (human) 18S rRNA and 28S rRNA
NA-No Amplification
ND-Not Done

Supplemental Table S4. TBDCapSeq analysis of acute Lyme disease and babesiosis samples

Sample	Sample type	# of raw reads	# of input reads	# of reads mapped to B31^	% of mapped reads	# of reads mapped to 16S-23S rRNA^	Non-16S-23S rRNA reads mapped	# of non ribosomal reads mapped to *B. microti*
LYM-851	Extraction control	2,392,041	1,825,793	14	0.001	14	0	0
LYM-900	Lyme disease-EM$	2,999,782	2,311,272	14	0.001	14	0	0
LYM-901	Lyme disease-EM	3,499,493	2,658,689	33	0.001	33	0	0
LYM-902	Lyme disease-EM	3,673,875	2,734,197	9	0.000	9	0	0
LYM-903	Lyme disease-EM	2,587,057	1,988,271	10	0.001	10	0	0
LYM-905	Lyme disease-EM	2,522,778	1,921,627	36	0.002	36	0	0
LYM-906	Lyme disease-EM	1,978,756	1,480,265	8	0.001	8	0	0
LYM-907	Lyme disease-EM	2,583,643	1,969,052	0	0	0	0	0
LYM-908	Lyme disease-EM	2,915,520	2,215,750	10	0.0005	10	0	0
LYM-909	Lyme disease-EM	2,747,290	2,118,051	0	0	0	0	0
LYM-910	Lyme disease-EM	2,885,228	2,198,919	9	0.0004	9	0	0
LYM-911	Lyme disease-EM	2,476,977	1,888,135	0	0	0	0	0
LYM-918	Lyme disease-EM	6,907,453	5,199,859	2	0	2	0	0
LYM-922	Lyme disease-EM	3,670,664	2,759,222	0	0	0	0	0
LYM-920	babesiosis	6,974,578	5,341,651	13	0.0002	13	0	863,208
LYM-924	babesiosis	261,359,660	233,945,597	621	0.0003	621	0	228,468,322
LYM-926	babesiosis	173,292,460	155,873,645	177	0.0001	177	0	152,509,752
Salmon sperm	control	2,811,546	1,377,421	69	0.005	69	0	0

Sample		# raw reads	# of input reads	# of reads mapped to B31	% of mapped reads	# of reads mapped to 16S-23S rRNA	Non-16S-23S reads mapped	plasmid
LYM-904*	Lyme disease-EM	2,385,536	1,802,362	69	0.004	9	60	-
							14	cp32-3
							17	cp32-6
							6	cp32-7
							13	cp32-9
							10	lp56
LYM-912*	Lyme disease-EM	2,327,392	1,766,086	72	0.004	15	57	-
							1	cp32-3
							12	cp32-4
							11	cp32-9
							7	lp28-3
							15	lp36
							8	lp38
							3	cp26

*Samples LYM-904 and LYM-912 were the only samples with authentic reads mapping to *B. burgdorferi*. The last two columns on the right show the read distribution among the genomic segments of *B. burgdorferi*. Both samples had 0 reads mapped to *B. microti*. ^all reads mapped to B31 16S or 23SrRNA represented non-*Borrelia* reads $EM-erythema migrans

Supplementary Table S5. Strains selected for probe design.

Agent	Strain
Borrelia burgdorferi	B31, N40, 297
Borrelia mayonii	MN14-1420
Borrelia miyamotoi	CT14D4
Babesia microti	RI
Anaplasma phagocytophilum	HZ
Ehrlichia muris (eauclairensis)	Wisconsin_h
Ehrlichia chaffeensis	Jax
Rickettsia parkeri	Grand Bay
Rickettsia rickettsii	Hlp#2
Powassan virus	LB, LI1
Heartland virus	Patient 1
